# Genome-Wide Association of Heroin Dependence in Han Chinese

**DOI:** 10.1371/journal.pone.0167388

**Published:** 2016-12-09

**Authors:** Gursharan Kalsi, Jack Euesden, Jonathan R. I. Coleman, Francesca Ducci, Fazil Aliev, Stephen J. Newhouse, Xiehe Liu, Xiaohong Ma, Yingcheng Wang, David A. Collier, Philip Asherson, Tao Li, Gerome Breen

**Affiliations:** 1 Institute of Psychiatry, Psychology and Neuroscience, MRC SGDP Centre, King’s College London, United Kingdom; 2 MRC Integrative Epidemiology Unit, University of Bristol, Bristol, United Kingdom; 3 Department of Actuarial Sciences and Risk Management, Faculty of Business, Karabuk University, Karabuk, Turkey; 4 Mental Health Center, West China Hospital, Sichuan University, Sichuan, People’s Republic of China; 5 Psychiatric Laboratory, State Key Laboratory of Biotherapy, West China Hospital, Sichuan University, Sichuan, People’s Republic of China; 6 Lilly UK, Erl Wood Manor, Windlesham, Surrey, United Kingdom; 7 Department of Psychiatry, West China Hospital, School of Medicine, Sichuan University, Sichuan, People’s Republic of China; Johns Hopkins University Bloomberg School of Public Health, UNITED STATES

## Abstract

Drug addiction is a costly and recurring healthcare problem, necessitating a need to understand risk factors and mechanisms of addiction, and to identify new biomarkers. To date, genome-wide association studies (GWAS) for heroin addiction have been limited; moreover they have been restricted to examining samples of European and African-American origin due to difficulty of recruiting samples from other populations. This is the first study to test a Han Chinese population; we performed a GWAS on a homogeneous sample of 370 Han Chinese subjects diagnosed with heroin dependence using the DSM-IV criteria and 134 ethnically matched controls. Analysis using the diagnostic criteria of heroin dependence yielded suggestive evidence for association between variants in the genes *CCDC42* (coiled coil domain 42; *p* = 2.8x10^-7^) and *BRSK2* (BR serine/threonine 2; *p* = 4.110^−6^). In addition, we found evidence for risk variants within the *ARHGEF10* (Rho guanine nucleotide exchange factor 10) gene on chromosome 8 and variants in a region on chromosome 20q13, which is gene-poor but has a concentration of mRNAs and predicted miRNAs. Gene-based association analysis identified genome-wide significant association between variants in *CCDC42* and heroin addiction. Additionally, when we investigated shared risk variants between heroin addiction and risk of other addiction-related and psychiatric phenotypes using polygenic risk scores, we found a suggestive relationship with variants predicting tobacco addiction, and a significant relationship with variants predicting schizophrenia. Our genome wide association study of heroin dependence provides data in a novel sample, with functionally plausible results and evidence of genetic data of value to the field.

## Introduction

Substance dependence, such as heroin addiction, can have a devastating impact on the lives of the affected individuals, their families and the wider society. According to the World Health Organization (UNODC, World Drug Report 2012; www.who.int), heroin use has increased two to three fold since the 1980s and the ensuing health concerns are severe; the risk of death in drug users is 20 to 30 times greater than in non-drug users, mostly from overdose or acquired infections. Heroin produces strong euphoric effects and addiction can develop rapidly in vulnerable individuals. Dependence on the drug represents a chronic and relapsing condition, characterized by compulsive consumption, craving, tolerance, withdrawal symptoms and negative behavioral effects. Although dependency is frequently a culmination of complex interactions between behavioral, cognitive, and physiological factors, genetic factors can contribute 30–80% of the liability to risk [1,2]. The combination of findings from genetic studies, model organisms and molecular studies in humans has led to the hypothesis of a biological underpinning to heroin dependence, with particular emphasis on the role of the central nervous system [3]. This has inevitably led to an interest in genes encoding molecules in neural systems; these represent biologically plausible candidate genes and those involved in reward-processing, cognition, stress and anxiety have been studied most intensively [4]. As opioid receptors are critical for modulating the euphoric effects of the drug, variants in the genes encoding the opioid receptors have been tested extensively and there is good evidence in support of the A118G polymorphism in the mu opioid receptor gene (*OPRM1*) [5,6], albeit with mixed results. A recent meta-analysis has conducted a thorough analysis of this polymorphism, by defining uniform phenotypes across a range of addictive substances, including heroin, and reported a modest protective effect of the G allele in European populations [7]. Other neural systems have also been tested, including the dopaminergic, glutamatergic, GABAergic, and serotonergic [8–10] systems. The candidate gene approach relies on a prior hypothesis of functionally plausible genes, yet has yielded few robust or replicated findings, being limited by a number of flaws including a neglect of the false positive rate inherent in genetic studies. One can posit that other biological systems, hitherto untested, may be relevant and indeed provide information related to mechanisms underlying heroin addiction; the genome-wide approach which is agnostic to prior hypotheses offers the opportunity of finding such novel genetic variants.

Genome wide association studies (GWAS) testing heroin addiction [11,12] and opioid dependence [13,14] have produced evidence of the role of novel loci and even replicated evidence from candidate gene studies. In the very first GWAS on heroin addiction, [11], testing 104 methadone-maintained former heroin addicts of Caucasian ethnicity and 101 matched controls, the authors reported suggestive association with *GABRA3* (gamma-aminobutyric acid, receptor subunit alpha 3), a candidate gene for heroin addiction. Despite the small sample size, one marker (rs965972; chr1q31.2) survived correction for multiple testing, however it was not located near any gene. Building on these early findings, the authors expanded the study by increasing the sample size as well as the number of markers tested [12]; 325 ethnically mixed, methadone-stabilized former heroin addicts were compared to 250 control individuals using a 100K Affymetrix array. Some overlap was observed between the two studies, whereby the top markers were located on chromosome 1q23, albeit about 30kb apart. In the African-American sub-sample (125 cases; 100 controls), the most significant SNP (rs950302) was located in the gene *DUSP27* (cytosolic dual specificity phosphatase 27), with point-wise significance (p = 0.0079) for association with heroin addiction vulnerability.

Two recent GWAS are of particular relevance, one because of the larger sample size and the additional analyses for risk pathways [13], while the second one used exposed and non-exposed controls [14]. Gelernter et al [13] performed a well-designed study utilizing an initial discovery sample of 5697 individuals, followed by two replication stages: stage 1, N = 4063 participants; stage 2, N = 2549 participants, all satisfying the criteria for opioid dependence. The increase in sample size was sufficient to produce results that were replicated across the different phases and interestingly, that were functionally relevant to the phenotype. Association analyses produced population-specific variants for the analyses using case-control status, symptom count and meta-analyzed results of all three phases. The African-American sample yielded the most significant results with variants in *KCNG2* (potassium voltage-gate channel modifier subfamily G member 2). Subsequent pathway analyses captured the role of subthreshold results to reveal two potentially functional pathways, calcium signaling and synaptic long term potentiation. Neurotransmitter signaling plays a key role in drug dependence [3,15]; *CAMK2B* (calcium/calmodulin dependent protein kinase II beta) was shown to be a hub molecule in pathways relevant to drug addiction [16], whereas long term potentiation could modulate heroin relapse through the glutamate receptor *NR2B* (NMDA2b-containing receptor) [17]. The groundbreaking study by Nelson et al [14] utilized a novel and highly valid design, the comparison of exposed and non-exposed controls with affected individuals. Most genetic studies in addiction tend to use unexposed controls which are useful for assessing dependence on drugs but tend to lead to reduced power when analyzing intermediate or later stages of addiction. Comparison of opioid-dependent subjects with opioid misusers, namely those individuals who had not progressed to dependence, revealed a protective role of variants in the gene *CNIH3* (cormichon family AMPA receptor auxiliary protein 3). Identification of protective variants is useful as these could serve as biomarkers to prevent transition from opioid use to dependence, and thus help translational work.

The current study is the first genome wide study to test Han Chinese individuals for association with heroin dependence. The samples were hybridized to the Illumina HumanCoreExome-12v1_A microarray, developed to capture extensive genomic variation including rare single nucleotide variants and insertions/ deletions (indels). We assessed the data for risk variants as well as pathways that might be functionally relevant in heroin dependence. It would have been relevant to test exposed controls but these were not available hence the analysis was limited to dependence. Nonetheless, the results are of value due to the novelty of population tested as well as the results of the *post hoc* analyses, such as gene-based association tests, and polygenic risk scores.

## Materials and Methods

### Ethics statement

Written, informed consent was obtained from all participants and peripheral blood samples were collected for DNA extraction. The study was conducted in accordance with ethics approval granted by Internal Review Committees of King’s College London, UK (No. 103/02) and West China Hospital, for conducting genetic studies using the Chinese sample of cases and controls. The UK National Health Service (NHS) Research Ethics Review Committee approved the use of the sample for large scale genetic studies.

### Subjects

Our initial sample of 567 individuals was comprised of 398 heroin addicts and 169 controls, with a mean age of 26–31 years, as shown in Table 1. The participants were predominantly northern Han Chinese from Sichuan Province; these are a more homogeneous population than the southern Han. Chinese ethnic minorities were not included in the sample to reduce the likelihood degree of population stratification. The cases were recruited from inpatient clinics attached to two psychiatric hospitals in Chengdu (Southwest China) and were diagnosed with DSM-IV criteria for heroin dependence using a semi-structured clinical interview. This interview included questions on (i) age at first use of heroin and duration of use, (ii) the quantity of drug consumed over this period, (iii) whether the patient had abused other addictive substances such as alcohol, cocaine, cannabis, etc., (iv) co-morbidity of other psychiatric conditions. Those subjects who were abusing other substances or suffered from a major psychiatric illness such as recurrent major depression, schizophrenia or bipolar disorder were not included in the study. To corroborate patient interview, case notes were examined and a family member was also used as an informant. As the abuse of drugs other than heroin is uncommon is Southwest China, it was possible to include most subjects interviewed. However, there were some subjects who admitted to excessive alcohol use and these were excluded. The control sample was recruited from college staff, medical students and acute medical inpatients in a general hospital, none of whom had any neurological or psychiatric disorders or a family history of psychiatric or addictive disorders. The control participants did not undergo a formal psychiatric interview but were asked if they had ever been told by a doctor that they suffered from a mental or neurological illness, or had been prescribed drugs or admitted to hospital for such an illness.

**Table 1:** Demographic information on the Han Chinese sample used in the study.

	Cases		Controls	
	*Males*	*Females*	*Males*	*Females*
Total number	302	96	72	97
Mean age	27.6±5.19	26±6.77	29.9±10.7	31±10.9

### Genotyping and quality control

Genomic DNA was extracted from blood samples using standard phenol-chloroform procedures and was initially quantified using spectrophotometry. Prior to hybridization to the chip, all samples were re-quantified using pico-green fluorimetry and DNA quality was assessed using standard gel electrophoresis techniques; this ensured that only samples of high quality DNA were used. Automated procedures were used to hybridize the cases and controls to the HumanCoreExome-12v1_A Beadchip (Illumina Inc., San Diego CA, USA) and scanned on the Illumina HiScan platform, using standard protocols. This particular chip comprises of 547644 markers, including all the tag markers (264,909 markers) on the HumanCore beadchip, over 240,000 markers of the HumanExome chip as well as several rare variants.

### Quality control

Genotyped data was first assessed in GenomeStudio using the GenCall algorithm (Illumina), however as the program is better at examining common variants, rare variants were then assessed using zCall [18], a rare-variant caller specially designed for microarrays. In accordance with the analytic pipeline developed in-house [19], the data from the initial genotype calling was subjected to further stringent quality control in PLINK [20] and PLINK2 [21]. The resulting dataset taken forward to imputation analysis consisted of 263084 autosomal SNPs, with MAF >5%, call rate >99% and which did not deviate from Hardy-Weinberg with *p*>1x10^-5^. A large number of SNPs were eliminated during this QC stage, as they were not polymorphic in the Chinese sample or were too rare; setting the MAF cutoff at 5% enabled us to include more of the common variants. Individuals with call rates of >99%, gender consistent with the heterozygosity of X chromosome SNPs and with genome-wide SNP heterozygosity within 2SD of the sample mean were retained, leaving a total of 504 samples (370 cases and 134 controls) available for genetic analyses.

### Case-control association

Following QC, 370 cases were compared with 134 controls for association using logistic regression, adjusting for two ancestry-informative covariates, in PLINK [20]. Power analysis using 400 cases and 150 controls with the online calculator, the Center for Statistical Genetics (CaTS) power calculator program (http://csg.sph.umich.edu//abecasis/cats/), indicates that at a significance level of p = 0.0025, such a sample size has 60% power to detect common alleles with frequency of 0.5. (Figure A(i) in S1 File), and expectedly has reduced power to detect alleles of lower allele frequency (Figure A(ii) in S1 File). To account for multiple testing issues, the standard GWAS significance threshold of α = 5x10^-8^ [22] was used. Annotation of gene names for the SNPs was conducted using SeattleSeq http://snp.gs.washington.edu/SeattleSeqAnnotation137/) [23]. For those markers not annotated by the software, gene names were searched on the UCSC Genome Browser, build 37/hg19 (http://genome.ucsc.edu/). For the 100 most significantly associated SNPs from logistic regression, flanking regions of 50kb on either side were also searched. Finally, in order to identify independent association signals in our data, we applied the clumping procedure in PLINK2 [21], taking all SNPs associated with our phenotype with *p*≤0.001, termed index SNPs, and identifying SNPs that are in LD with the index SNP (r^2^ > 0.5) within a sliding window of 250kb.

### Imputation

Genotypes were imputed to NCBI build 37 using Phase 1 of the 1000 Genomes reference data and selecting for the Asian population for ethnicity, as implemented on the Minimac server (http://imputationserver.sph.umich.edu/) [24]. Following imputation, duplicate IDs corresponding to triallelic SNPs were removed. In accordance with our imputation pipeline, we removed SNPs with MAF < 0.01, imputation quality R^2^ < 0.9 and average call rate of <0.95. The imputation analysis produced a post-imputation analytic sample of 4,009,606 SNPs which was subjected to further QC. The imputed data, after removal of the major histocompatibility complex (26–33 Mb on chromosome 6), and pruning, was used to calculate two ancestry-informative covariates, using Multidimensional Scaling. These ancestry-informative covariates were used to adjust for any population structure.

### Gene based association testing

We used VEGAS2 (Versatile Gene-based Association Study) [25], an updated version of VEGAS [26], to calculate gene-based *p*-values from the association results. This online software tool uses population-based estimates for linkage disequilibrium (LD) and SNP-based *p*-values from GWAS, to identify significant deviations from expected *p*-value distributions within genes, under the null. In VEGAS2, this information is derived using the 1000 Genomes phase 1 data, enabling improvements in the LD estimates and allows analysis of X-chromosome. The analysis provides a statistic for gene-based results that is sensitive to gene length and recombination hotspots, as well as identifying genes in which there are multiple independent signals across cases, each of which individually may not reach genome-wide significance. For our analysis we used the Asian reference data, selecting the Chinese reference panel to estimate LD within genes and calculate gene-based empirical tests of association. Margins of +/- 50kb were set for LD estimates and all chromosomes were included except chromosome Y and mitochondrial genes.

### In silico replication

Possible replication of our association results was assessed using summary statistics for 1331 individuals of African-American ancestry and 1814 individuals of European-American ancestry, tested for genome-wide association with opioid dependence by Gelernter and colleagues [13]. The study samples had been ascertained at five different US sites and all subjects had satisfied the DSM-IV criteria of opioid dependence with the Semi-structured Assessment for Drug Dependence and Alcoholism [27] and provided written, informed consent as described in Gelernter et al [13]. GWAS had been performed on the Illumina HumanOmni1-Quad v1.0 chip. For our replication assessment, we prioritized the top 100 SNPs from our imputed association results and limited our replication to SNPs located within or close to the top genes, *CCDC42* and *BRSK2*. Prior to the assessment, we checked for concordance of marker locations between the two datasets. A total of 49 SNPs were examined for replication, 24 markers in *CCDC42* and 25 markers in *BRSK2*. In addition to the SNP-based replication, we were interested in assessing the association status in our data of candidate genes that had been previously tested in Chinese populations. A literature search of previously published studies provided us with a list of candidate genes, noted in Table C(i) in S2 File.

### Functional and pathway assessment

Pathways were extracted from MSigDB v5.2 canonical pathways (CP) and Gene Ontology (GO) datasets; MSigDB [28] is distinguished for having the largest collection of gene sets, derived from diverse gene set sources. Only pathways containing 10–1000 genes were included, yielding a total of 7111 pathways (1309 CP, 5802 GO) for the analysis. We compiled gene sets in our association data using a 35kb upstream and 10kb downstream window to include gene regulatory regions and MAF ≥ 0.05. The genes were encoded by ENSEMBL identifiers (release 75, genome assembly h19). Pathways were assigned competitive p-values using MAGMA v1.05 [29] which assesses whether a pathway is more associated with a trait than other pathways, and takes into account linkage disequilibrium (LD). The reference data used for LD was the Southern Han Chinese subset (CHS) of 1000 genomes phase III data [30]. The gene and pathway p-values were adjusted using Benjamini-Hochberg FDR procedure [31] to obtain q-values. In silico tissue specific expression of the top genes from the association and VEGAS2 analyses, was examined using the freely available online database, Genotype-Tissue Expression (GTEx) Portal (http://www.gtexportal.org/).

### Polygenic risk scores

Genome-wide association studies in neuropsychiatric disorders tend to produce small effect sizes; even the most significantly associated markers tend to have small effects, with odds ratios (OR) in the range of 0.8–1.2. These small effect sizes, and the resultant lack of power, mean that the majority of disease associated SNPs fall below genome-wide significance; markers rejected by GWAS can be combined into quantitative scores to examine the combined effects of the variants [32,33]. We calculated polygenic risk scores (PRS) using PRSice [34]. PRSice calculates the best-fit PRS across 10,000 thresholds (from P_T_ = 0.0001 to P_T_ = 0.5 by increments of 0.00005) by regressing phenotype on score and two ancestry informative covariates. To calculate PRS in this study, we used publicly available GWAS results as base datasets; these are the five most recent analyses by the Psychiatric Genomics Consortium (PGC)—schizophrenia, depression, ADHD, autism and bipolar disorder [35–39]–and four phenotypes from the Tobacco And Genetics Consortium—ever smoked, quantities of cigarettes smoked, former smoker and age at starting smoking [40].

## Results

### Association analysis

**Fig 1:**
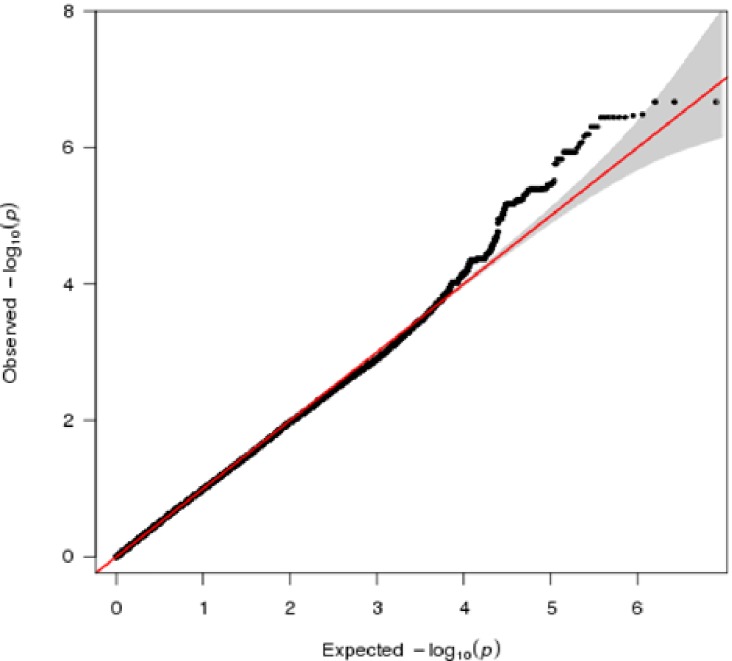
Quantile-quantile plot for test statistic inflation, lambda median = 1.026.

Following quality control and imputation, data was available on 4,009,606 markers genome-wide, in 504 individuals. We performed association testing between 370 cases and 134 controls, adjusting for population structure using two ancestry-informative dimensions generated using multidimensional scaling. We found no evidence for residual population structure after using these covariates, as shown in the QQ plot in Fig 1 (GWAS λ _median_ = 1.026). In this study, the most significant markers demonstrated p-values of 10^−7^ (Table 2). Association testing identified suggestive association between heroin dependence and several markers located within genes that may be of relevance to heroin dependence; the 25 most associated independent markers are listed in Table 2. Among these were several markers located on chromosome 17p13.1; indeed the three most associated genotyped SNPS were all located either in or near the gene *CCDC42* (coiled coil domain containing 42) on chromosome 17 (see Table A in S2 File for details on the 100 most associated genotyped SNPs). Many of the top markers are located within or close to the genes *CCDC42*, *BRSK2* (BR serine/threonine kinase 2), *ZNF546* (zinc finger protein 546), *CHIT1* (chitinase 1) and *PPP1R12B* (protein phosphatase 1, regulatory subunit 12B) and *NEK1* (NIMA-related kinase 1).

**Table 2:** Top 25 results from the pre-imputation analysis of case-control association with heroin dependence, after implementation of clumping in PLINK to identify relatively independent signals. Genes are mapped based on chromosome and base pair position, using the program SeattleSeq.

CHR	SNP	BP	A1	MAF	Gene	OR	P
17	rs4791746	8626357	T	0.3867	Unmapped	0.4642	2.229e-07
17	rs2288156	8644854	T	0.1729	CCDC42	0.3905	2.819e-07
17	17:8631468	8631468	C	0.3799	Unmapped	0.4694	3.788e-07
8	rs4739179	78785992	G	0.3477	Unmapped	0.5038	3.62e-06
11	rs1881509	1425605	G	0.4092	BRSK2	0.4947	4.148e-06
8	8:78718310:A_AC	78718310	I	0.3525	Unmapped	0.5099	4.925e-06
11	11:1421138:T_TGG	1421138	I	0.4102	Unmapped	0.4998	5.255e-06
7	rs78158938	36786796	A	0.09766	Unmapped	0.203	6.159e-06
20	rs6022774	52431105	A	0.4971	Unmapped	0.512	1.067e-05
1	rs1417150	203196757	T	0.08887	CHIT1	0.3534	1.269e-05
4	rs9917891	9614633	C	0.02344	Unmapped	0.133	1.299e-05
17	rs9894347	8646158	C	0.498	CCDC42	0.5309	1.352e-05
3	rs17422129	82969622	C	0.3145	Unmapped	0.5235	1.601e-05
20	rs6095949	49061728	G	0.498	Unmapped	1.889	2.082e-05
2	rs13426854	240845694	T	0.03418	Unmapped	0.2091	2.732e-05
18	rs8085967	52654114	A	0.09082	Unmapped	0.3811	2.972e-05
10	rs7916242	54048234	G	0.3809	PRKG1	0.5441	3.159e-05
11	rs11532013	98364555	G	0.08398	Unmapped	0.3793	3.342e-05
13	rs9587328	107911258	A	0.1064	FAM155A	0.3929	3.5e-05
3	rs3732377	39138840	G	0.1904	GORASP1	0.4711	3.71e-05
5	5:81799177	81799177	C	0.04492	Unmapped	0.2743	3.752e-05
7	7:83000350:GGTGC	83000350	D	0.09766	SEMA3E	0.4033	3.967e-05
7	rs12111869	82998022	T	0.03711	SEMA3E	0.4033	3.967e-05
7	rs4368921	131343761	G	0.4043	Unmapped	1.955	4.142e-05
3	3:38939207:TA_T	38939207	R	0.334	SCN11A	0.5295	4.254e-05

**Fig 2:**
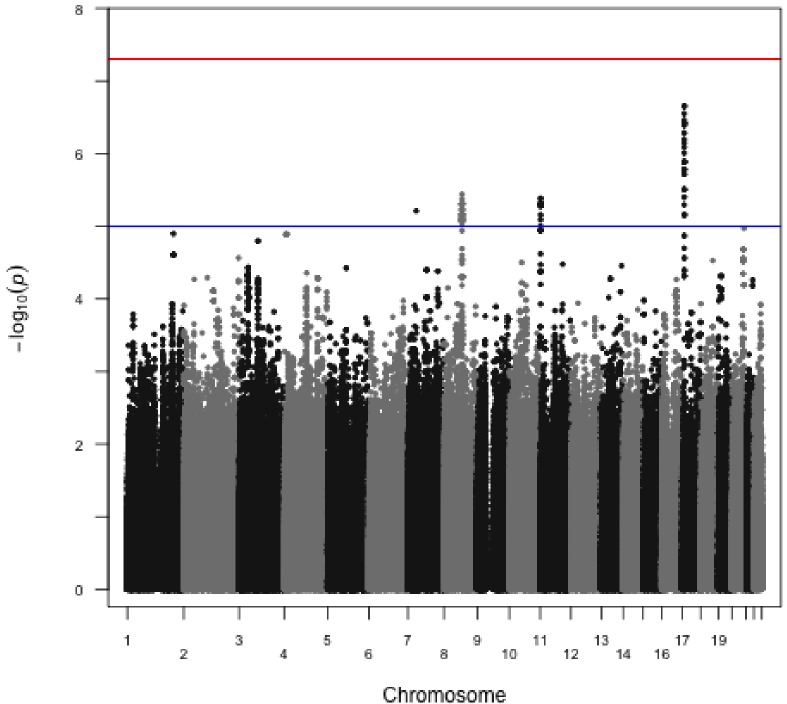
Manhattan plot showing post-imputation genome-wide association results. Data for chromosomes X and Y was removed prior to imputation. Analysis highlighted three main chromosomal regions of association. The blue line denotes a p-value cutoff of 10^−5^ (suggestive significance) and the red line is at p-value = 5x10^-8^ (genome-wide significance).

We identified three distinct clusters of associated SNPs, on chromosomes 17, 11 and 8 (Fig 2). The two smaller clusters of markers on chromosome 11p15.5 and chromosome 8q21.12 yielded suggestive association with p < 10^−6^ and 19 markers with p < 10^−7^ were located either within or close to *CCDC42* on chromosomes 17 (Table A in S2 File). The regions on chromosomes 8 and 11 contained a density of markers with p≤3.5 x 10^−5^ (Table A in S2 File). The genes *PXDNL* (peroxidasin-like), *PCMTD1* (protein-L-isoaspartate (D-aspartate) O-methyltransferase domain containing 1) and *ARHGEF10* (Rho guanine nucleotide exchange factor (GEF) 10) all localized to the region on chromosome 8. The markers on chromosome 11 predominantly localized to the gene *BRSK2*.

### Gene based association testing

Applying VEGAS2 [25] to the results of our genome-wide association results identified two genes *CCDC42* and *SPDYE4* meeting the significance threshold of α = 2.85x10^-6^ (Table 3), as suggested by Liu et al [26]. This threshold is likely to be overly conservative, meaning that the data provides good evidence for an association between these genes and heroin dependence; it should be noted that the two genes are located close to each other and may represent a single association signal. The VEGAS2 output represents non-significant regions from GWAS attaining significance in gene-based analysis; this is consistent with multiple relatively independent causal loci within the same gene. This is supported by multiple relatively independent signals in *CCDC42* after clumping.

**Table 3:** The top 25 results from the gene-based analysis using VEGAS2. At the recommended significance threshold of P-value = 10^−6^, the genes *CCDC42* and SPDYE4 demonstrate significant results.

Gene	nSNPs	Start	Stop	Gene Pvalue	TopSNP	TopSNP-pvalue
CCDC42	181	8583245	8698154	1.00E-06	rs4791746	2.23E-07
SPDYE4	182	8606423	8711877	1.00E-06	rs4791746	2.23E-07
SMAD1	192	146352950	146530325	8.60E-05	rs28480984	5.21E-05
SGPL1	264	72525703	72690932	0.00018	rs12782980	6.56E-05
MAP4K2	48	64506608	64620713	0.000227	rs490980	0.0001452
CD7	9	80222745	80325480	0.000244	rs8072762	0.0002111
SMAD1	129	146368200	146474132	0.000282	rs76068476	0.0001389
SF1	63	64482075	64596316	0.000296	rs490980	0.0001452
ADD3	30	111655316	111818139	0.000408	rs10466193	0.0007459
PCBD1	125	72593264	72698543	0.000411	rs10509327	0.0003889
MEN1	48	64520985	64628766	0.000413	rs490980	0.0001452
IFITM5	73	248200	349526	0.000433	rs11246088	0.0002424
TBATA	255	72480994	72595157	0.000484	rs12782980	6.56E-05
IFITM2	76	258106	359410	0.000485	rs11246088	0.0002424
PYGM	70	64463860	64578187	0.000509	rs490980	0.0001452
FAM21C	91	46172647	46338412	0.000559	rs138643555	0.000144
KDELC2	140	108292832	108419159	0.000611	rs10749917	0.001527
SECTM1	22	80228899	80341921	0.000700	rs8072762	0.0002111
MIR1976	125	26831032	26931084	0.000725	rs737465	0.0001625
KAAG1	116	24307130	24408512	0.000731	rs6940827	0.0006796
RASGRP2	72	64444382	64562928	0.000787	rs490980	0.0001452
SPATA31D1	21	84553686	84660171	0.000829	rs149183278	0.0006814
UTS2R	56	80282200	80383370	0.000960	rs8072762	0.0002111
ZFAND4	287	46060948	46218261	0.001080	rs138643555	0.000144
LGI3	43	21954342	22064344	0.001100	rs6557826	0.0005859

### In silico replication

Assessment of replication using summary statistics demonstrated no evidence of replication; results are presented in the supplementary data, Table B(i) and (ii) in S2 File. We present comparison of the two genes *CCDC42* and *BRSK2* with the data for the two different populations, African-American and European-American. In general, the replication samples demonstrated p-values > 0.1 compared to p-values <10^−5^ in our dataset, for the selected markers. When examining the association status of candidate genes, we did not observe association with variants in the selected candidate genes. The results are presented in the supplementary data; in Table C(i) in S2 File we present the candidate gene list compiled using previously published studies and Table C(ii) in S2 File shows results in our study, highlighting p-values of the most associated SNPs in these genes.

### Pathway analysis

Functional pathway analysis using MSigDB yielded top pathways related to regulation of vacuolar transport, regulation of skeletal muscle contraction and cellular processes relevant for cell division, however none of the pathways demonstrated an empirically significant result. Table D in S2 File in the supplementary data lists the top ten pathways, with the empirical P-value for the pathway. We examined the expression patterns of some of the top genes, using GTEx Portal (www.gtexportal.org). The gene *CCDC42* is the top gene in the association analysis as well as in the gene-based analysis; expression patterns show that it is primarily expressed in the testes with no observable expression levels in any other tissue. The gene *BRSK2* is expressed in several parts of the brain, with expression levels being highest in cerebellum tissues, followed by somewhat lower expression in the hippocampus and the hypothalamus. According to the database, there is some expression in the pancreas and the pituitary. Finally, *ARHGEF10* shows expression mainly in tissues derived from the tibial nerve and the lung with smaller amounts of expression in a range of other tissues, including the brain.

### Polygenic risk scoring

We used PRSice to calculate polygenic risk scores for liability to a number of psychiatric traits, and evaluated their ability to predict heroin addiction (Table 4). The base data included GWAS for smoking and related behaviors and datasets for diseases known to be comorbid with addiction, for example, schizophrenia and ADHD. We found significant evidence that genetic risk of schizophrenia could predict heroin dependence, (p = 0.0007) at P_T_ = 0.0085. This is likely in part explained by the relatively higher power of the schizophrenia GWAS, compared to the other psychiatric traits investigated, leading to improved identification of any shared genetic factors between heroin dependence and any generalized liability to psychiatric disorders [41]. We did not find evidence for a genetic overlap between heroin dependence and any of the other GWAS traits investigated here. The data from the Tobacco Addiction Genetics (TAG) Consortium may be the most relevant for the phenotype of heroin addiction; in a previous study [42], PRS for cigarettes per day had predicted the number of glasses of alcohol consumed per day and age at onset of smoking predicted age at regular drinking. In the current study, no smoking phenotypes studied (ever smoked, cigarettes per day, former smoker, age of onset for smoking) predicted heroin addiction, when using the suggested significance threshold of α = 0.001; instead, there was a suggestive relationship with ‘ever smoked’ and age at first cigarette. The proportion of variance in heroin use explained by polygenic risk score was very low or moderately low (Nagelkerke’s R2 = 0.001–0.06) for all the phenotypes tested (Figure B(i)-(iv) in S1 File).

**Table 4:** Results of Polygenic Risk Scoring.

Base Phenotype	Best P-value Threshold	P-value at best threshold	Variance Explained (Nagelkerke’s Pseudo R2)
ADHD	0.0015	0.014874	0.01748
Autism	0.00735	0.028201	0.01417
Bipolar Disorder	0.00005	0.020131	0.01587
MDD	0.00025	0.087017	0.00852
Schizophrenia	0.0085	**0.000695**	0.03386
Cigarettes per Day	0.00005	0.150855	0.00597
Age at onset for smoking	0.215	0.037109	0.01261
Ever smoked	0.2271	0.020949	0.01557
Former smoker	0.0004	0.074126	0.00933

## Discussion

Our study was aimed at conducting a genome-wide association study (GWAS) to identify genetic variants that may play a role in heroin dependence and, with the aid of bioinformatics tools, to assess their expression patterns as well as functional pathways involved. The GWAS was performed using an ethnically homogeneous sample of Han Chinese origin; examination for presence of possible population substructure confirmed homogeneity of our sample of cases and controls. Case-control association analysis with 370 cases and 134 control individuals did not identify any genetic markers reaching genome-wide significance, as may be expected considering the relatively small sample size. Nonetheless, our gene based association analysis with VEGAS2, which involves fewer tests, also supported the putative role of *CCDC42*, consistent with the SNP based genome-wide analysis. Following imputation, three distinct regions were highlighted, on chromosomes 17, 11 and 8; the four most associated genes in these regions were *CCDC42*, *BRSK2*, *CHIT1* and *ARHGEF10*. The top ranked gene was *CCDC42* but little is known about the functional role of *CCDC42*, other than that it interacts with the transcriptional repressor *ZBTB1* (zinc finger and BTB domain containing 1), which in turn is functionally important for chromatin remodeling [43] (www.ncbi.nlm.nih.gov); this relationship suggests that *CCDC42* might be involved in epigenetic processes. A more intriguing functional relationship would be its involvement in immunological processes, as suggested by the association of *CCDC42* with Behcet’s disease [44]. Characterized by recurrent inflammatory attacks, Behcet’s disease affects orogenital mucosa, eyes, joints as well as the nervous system and the gastrointestinal tract [45]. A number of immunoregulatory pathways have implicated in Behcet’s disease; if *CCDC42* belonged to any of these pathways, it might explain its role in both Behcet’s disease and heroin addiction, as heroin use is often associated with increased presence of infectious diseases. Our gene-based analysis also yielded significant results with *CCDC42* as well as the neighboring gene, *SPDYE4* (Speedy/RINGO cell cycle regulator family member E4) of which little is known in terms of function.

The second of the top genes, *BRSK2* is one of a pair of kinases (BRSK1/2) highly expressed in the mammalian brain [46], in particular the cerebellum, hippocampus and hypothalamus tissues (www.gtexportal.org). The enzyme encoded by *BRSK2* localizes to presynaptic sites and modulates structural and functional maturation of synapses; indeed functional studies in mice indicate that the serine/threonine kinases (also known as SAD kinases) are critical for specification of axons and axonal development, and for playing a critical role in cell proliferation, differentiation and cell death [47]. The absence of SAD kinases does not prevent axon formation but does compromise maturation of axon terminals and mutations in SAD orthologs led to presynaptic defects in *C*. *elegans* [48] and *Drosophila* [49]. Gene ontology processes associated with *BRSK2* include actin cytoskeleton reorganization, apoptotic signaling pathways and establishment of cell polarity (www.uniprot.org).

Another one of the top genes in our study, *ARHGEF10*, is similarly involved in several cellular and actin cytoskeleton processes. As a member of the large family of rho guanine-nucleotide-exchange factors (GEFs), *ARHGEF10* acts as a molecular switch in the regulation of signal transduction pathways [50], binding to G-protein coupled receptors to stimulate downstream binding with protein kinases to affect cell signaling and extracellular stimuli processed through Rho proteins to modulate the intracellular actin cytoskeleton and subsequently intracellular processing [51,52]. Genetic studies in dogs suggest its role in neuropathies; a mutation in the gene resulted in a severe form of juvenile-onset polyneuropathy, which bears clinical similarity to the group of neuropathies termed Charcot-Marie-Tooth disease in humans [53]. Though expressed in multiple tissues, it has higher expression in the spinal cord and dorsal root ganglion [54]. Another gene showing nominal association (p≤0.001), *RASGRP2* (RAS guanyl releasing protein 2), encodes a brain-enriched nucleotide that contains an N-terminal GEF domain and may also play a role in cell signaling. The emerging functional themes related to the top genes in our data appear to be cellular processing and actin cytoskeleton restructuring; these are also identified in molecular studies investigating alcohol and cocaine addiction [55] and are therefore consistent with molecular models of addiction. It is already known that chronic and dependent drug consumption is correlated with structural plasticity in relevant neural circuits [56] and that such experience-dependent plasticity is primarily driven by changes in the shape and number of dendrites and dendritic spines [57–60]. Chronic administration of opioids can lead to reduction in the spine density [61]. In addition to the drug-induced plasticity, regulation of the cytoskeleton alterations may be influenced by mutations in the genes encoding the cytoskeleton proteins [55].

When looking at the individual SNPs, it is likely that we did not find loci associated with heroin dependence at genome-wide significance due to our relatively small sample size. Whether the genes identified in our study can be considered to have any pathophysiological role in heroin abuse depends on replication of these results and future functional studies. Our limited replication assessment using summary data from a large GWAS in opioid dependence showed no evidence of replication at the SNP level. Similarly, when we looked at some of the common candidate genes [62–73], our results did not show significant association with any of these genes (Table C(i) and (ii) in S2 File). Nonetheless, among the SNP-based and gene-based results, there are individual genes implicated in addiction. *ARHGEF10* has been discussed above, and *GRIN3B* (glutamate receptor, ionotropic, N-methyl-D-aspartate 3B) has been shown to be differentially expressed in heroin addicts compared to controls and methadone-maintained addicts [74], and was suggestively associated in gene-based analysis (p = 0.003; top SNP p = 0.6.8^−5^).

## Conclusion

The strength of this study lies in the homogeneous and novel population sample, and one that is not confounded by addiction to other drugs of abuse. We have identified suggestive association with a number of functionally plausible genes that might play a role in heroin dependence. Although the genes did not reach genome-wide significance, their putative role in addiction has been implicated through a range of *post hoc* analyses, such as our analysis of polygenic risk scores which revealed suggestive evidence that smoking-related behaviours may be modestly predictive of heroin addiction. Specifically, we find suggestive association with age at first cigarette, and ‘ever smoked’, which may indicate an overlapping susceptibility to novelty-seeking behavior; this may be confirmed in the future using higher-powered GWAS. Other genetic studies using Han Chinese subjects have tested similar sample sizes, of about 300 individuals [74]. Collecting large scale samples is difficult for substance use disorders such as heroin addiction; nonetheless, in order to achieve robust results, whether at the SNP level, gene or pathway level, a substantially larger sample size, in the range of tens of thousands of individuals, is required. Acknowledging this need, the Psychiatric Genetics Consortium (PGC) for Substance Use Disorders has been set up and data from the current study has been offered to the PGC-SUD, which is collecting data from other GWAS studies on substance use. The large number of samples will achieve the level of statistical power required and will provide much needed information on the etiological basis of opioid dependence. In conclusion, we have presented the results of the first genome wide study of heroin dependence in a Chinese sample, identifying novel associations between addiction and variation within genes that will be tested for replication in a large collaborative effort.

## Supporting Information

S1 File Output from the power analysis calculator, CaTS (Center for Statistical Genetics), estimating power for 370 cases and 170 controls at significance level of 0.0025 and selected minor allele frequency of (i) 0.5 and (ii) at 0.1. The y-axis shows statistical power for a range of sample s (x-axis) **(Figure A)**. Polygenic risk scores, using data on smoking behaviors obtained from the GWAS by the Tobacco and Genetics Consortium, show suggestive prediction for heroin addiction **(Figure B)**.
(DOCX)

S2 File Results of the genome-wide association with heroin dependence showing the top 100 SNPs, following imputation with the 1000 genomes phase 1 dataset, selected for the Asian population. The imputation produced 4M SNPs with top results on chromosomes 17, 11 and 8 **(Table A)**. Results of *in silico* replication of results for markers in *BRSK2* using summary data from a GWAS in opioid dependence in two different populations, African American and European American. (i). *In silico* replication of results for markers in and around the top gene, *CCDC42*, using summary data from a GWAS in opioid dependence in African American and European American populations (ii) **(Table B)**. Table showing common candidate genes previously tested in Chinese samples. The table shows the top result in these published studies and the type of polymorphism yielding the result. This list was used to assess gene-level replication in our data (i). Gene-based replication using the list of genes compiled from previous published reports, as shown in Table B(i) (above) and assessing their association status in the results from the VEGAS2 analysis performed in the current study (ii) **(Table C)**. Top ten results of the pathway analysis using the MSigDB database. The p-value calculated for each pathway was adjusted using the Benjamini and Hochberg method to yield an empirical value. The number of genes in each pathway is noted **(Table D)**.
(DOCX)
