# Supplementary material for: Genome-Wide Association of Heroin Dependence in Han Chinese
Source: PLoS One. 2016 Dec 9;11(12):e0167388. doi: 10.1371/journal.pone.0167388 (PMC5147879; doi:10.1371/journal.pone.0167388)
Supplement: S1 File — The y-axis shows statistical power for a range of sample s (x-axis) (Figure A). Polygenic risk scores, using data on smoking behaviors obtained from the GWAS by the Tobacco and Genetics Consortium, show suggestive prediction for heroin addiction (Figure B). (DOCX) [file pone.0167388.s001.docx]

**S1 Figs A (i), (ii):** Output from the power analysis calculator, CaTS (Center for Statistical Genetics), estimating power for 370 cases and 170 controls at significance level of 0.0025 and selected minor allele frequency of (i) 0.5 and (ii) at 0.1. The y-axis shows statistical power for a range of sample s (x-axis).

(i)


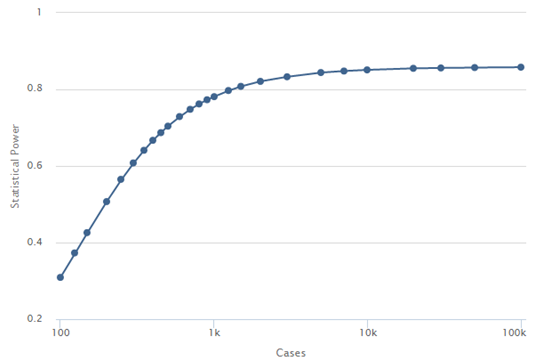


(ii)


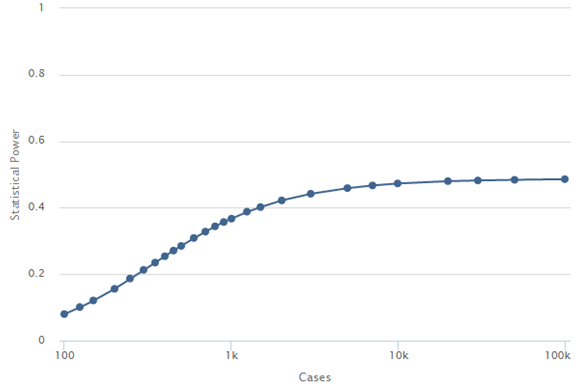


**S1 Fig B**: Polygenic risk scores, using data on smoking behaviors obtained from the GWAS by the Tobacco and Genetics Consortium, show suggestive prediction for heroin addiction

| Ever Smoked | Cigarettes per day |
| --- | --- |
| 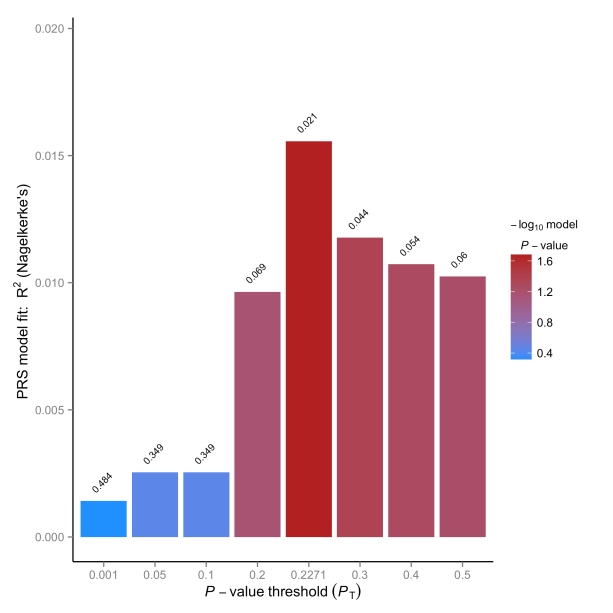 | 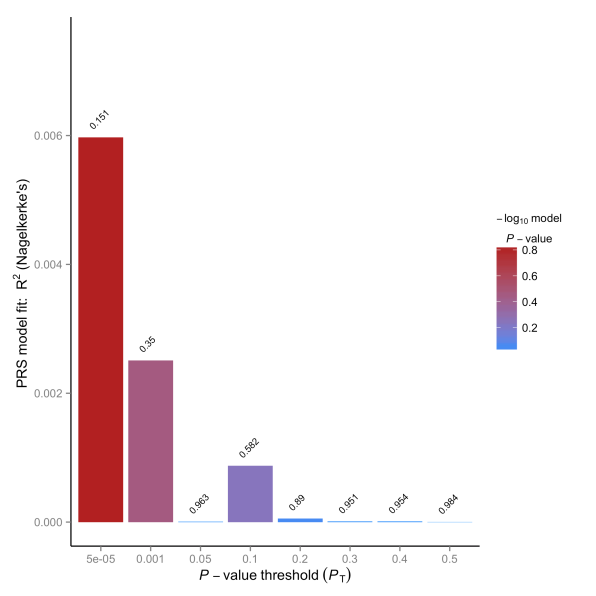 |
| Former Smoker | Age at Onset for Smoking |
| 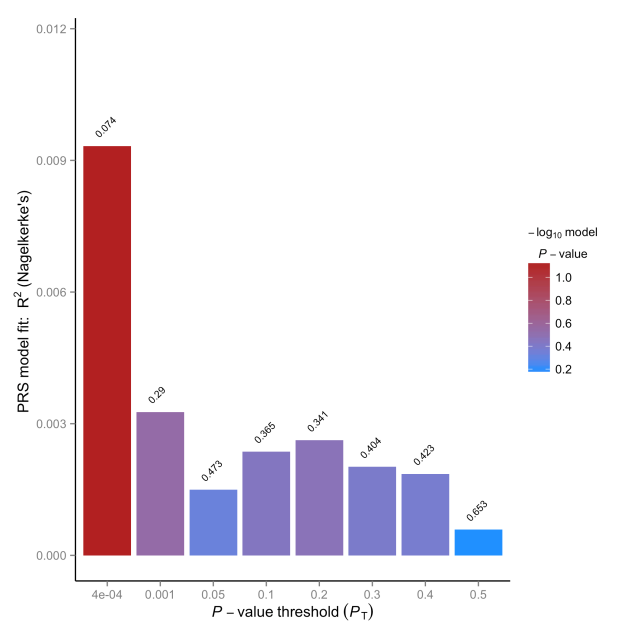 | 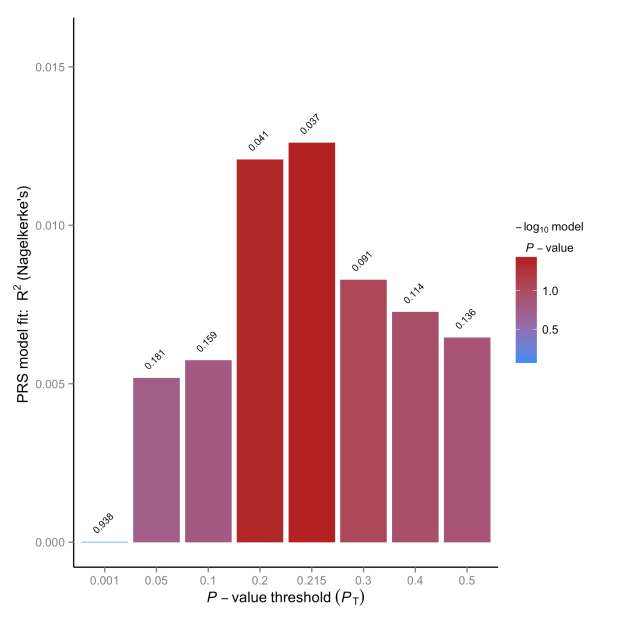 |
