# Supplementary material for: Genome-Wide Association of Heroin Dependence in Han Chinese
Source: PLoS One. 2016 Dec 9;11(12):e0167388. doi: 10.1371/journal.pone.0167388 (PMC5147879; doi:10.1371/journal.pone.0167388)
Supplement: S2 File — The imputation produced 4M SNPs with top results on chromosomes 17, 11 and 8 (Table A). Results of in silico replication of results for markers in BRSK2 using summary data from a GWAS in opioid dependence in two different populations, African American and European American. (i). In silico replication of results for markers in and around the top gene, CCDC42, using summary data from a GWAS in opioid dependence in African American and European American populations (ii) (Table B). Table showing common candidate genes previously tested in Chinese samples. The table shows the top result in these published studies and the type of polymorphism yielding the result. This list was used to assess gene-level replication in our data (i). Gene-based replication using the list of genes compiled from previous published reports, as shown in Table B(i) (above) and assessing their association status in the results from the VEGAS2 analysis performed in the current study (ii) (Table C). Top ten results of the pathway analysis using the MSigDB database. The p-value calculated for each pathway was adjusted using the Benjamini and Hochberg method to yield an empirical value. The number of genes in each pathway is noted (Table D). (DOCX) [file pone.0167388.s002.docx]

**Supporting Information Tables**

**S2 Table A**: Results of the genome-wide association with heroin dependence showing the top 100 SNPs, following imputation with the 1000 genomes phase 1 dataset, selected for the Asian population. The imputation produced 4M SNPs with top results on chromosomes 17, 11 and 8

| **CHR** | **SNP** | **BP** | **A1** | **MAF** | **AvgCall** | **R2** | **Gene** | **OR** | **P** |
| --- | --- | --- | --- | --- | --- | --- | --- | --- | --- |
| 17 | rs4791746 | 8626357 | T | 0.3892 | 0.9724 | 0.9159 | none | 0.4639 | 2.20E-07 |
| 17 | rs4791747 | 8626695 | A | 0.389 | 0.9724 | 0.916 | none | 0.4639 | 2.20E-07 |
| 17 | rs5019588 | 8626857 | T | 0.389 | 0.9723 | 0.9158 | none | 0.4639 | 2.20E-07 |
| 17 | rs7219141 | 8626010 | A | 0.3912 | 0.9711 | 0.911 | none | 0.4706 | 3.36E-07 |
| 17 | rs2288156 | 8644854 | T | 0.1731 | 0.9996 | 0.9985 | *CCDC42* | 0.3945 | 3.48E-07 |
| 17 | rs4791326 | 8631788 | G | 0.3895 | 0.9702 | 0.9102 | none | 0.4691 | 3.71E-07 |
| 17 | rs9303230 | 8630396 | G | 0.3897 | 0.967 | 0.8981 | none | 0.4691 | 3.71E-07 |
| 17 | rs9915929 | 8630420 | A | 0.3892 | 0.9674 | 0.8995 | none | 0.4691 | 3.71E-07 |
| 17 | . | 8631464 | C | 0.3896 | 0.9672 | 0.8984 |  | 0.4691 | 3.71E-07 |
| 17 | . | 8631468 | C | 0.3896 | 0.9672 | 0.8983 |  | 0.4691 | 3.71E-07 |
| 17 | . | 8631472 | C | 0.3896 | 0.9672 | 0.8983 |  | 0.4691 | 3.71E-07 |
| 17 | rs60363471 | 8642441 | C | 0.1697 | 0.9968 | 0.9803 | *CCDC42* | 0.3996 | 5.05E-07 |
| 17 | rs80051038 | 8641573 | T | 0.1696 | 0.9966 | 0.9791 | *CCDC42* | 0.3996 | 5.05E-07 |
| 17 | rs116694173 | 8642642 | T | 0.1697 | 0.9969 | 0.9806 | *CCDC42* | 0.3996 | 5.05E-07 |
| 17 | rs58558356 | 8639656 | A | 0.1699 | 0.994 | 0.9644 | *CCDC42* | 0.4037 | 6.53E-07 |
| 17 | rs73977403 | 8640012 | A | 0.17 | 0.9943 | 0.9661 | *CCDC42* | 0.4037 | 6.53E-07 |
| 17 | rs7215874 | 8628632 | T | 0.3919 | 0.9696 | 0.9069 | none | 0.4809 | 6.99E-07 |
| 17 | rs5019589 | 8626803 | A | 0.1632 | 0.984 | 0.9181 | none | 0.3967 | 8.47E-07 |
| 17 | rs7219393 | 8617731 | A | 0.377 | 0.984 | 0.9495 | none | 0.4858 | 8.98E-07 |
| 17 | rs112414721 | 8633714 | A | 0.1667 | 0.9874 | 0.9322 | *CCDC42* | 0.4073 | 1.02E-06 |
| 17 | rs4275897 | 8620609 | A | 0.3909 | 0.9905 | 0.9656 | none | 0.4905 | 1.19E-06 |
| 17 | rs4413002 | 8619809 | A | 0.3909 | 0.9926 | 0.9732 | none | 0.4905 | 1.19E-06 |
| 17 | rs4445935 | 8618603 | G | 0.3906 | 0.9951 | 0.9826 | none | 0.4905 | 1.19E-06 |
| 17 | rs4458042 | 8618608 | A | 0.3906 | 0.995 | 0.9824 | none | 0.4905 | 1.19E-06 |
| 17 | rs6503149 | 8620135 | C | 0.3907 | 0.9922 | 0.9717 | none | 0.4905 | 1.19E-06 |
| 17 | rs7208014 | 8619029 | G | 0.3906 | 0.9942 | 0.9795 | none | 0.4905 | 1.19E-06 |
| 17 | rs7223367 | 8618791 | G | 0.3892 | 0.9933 | 0.9758 | none | 0.4905 | 1.19E-06 |
| 17 | rs7225030 | 8618763 | A | 0.3906 | 0.9949 | 0.9818 | none | 0.4905 | 1.19E-06 |
| 17 | rs7225052 | 8618804 | A | 0.3892 | 0.9932 | 0.9755 | none | 0.4905 | 1.19E-06 |
| 17 | rs1975108 | 8615593 | A | 0.3912 | 0.9977 | 0.9919 | none | 0.4953 | 1.51E-06 |
| 17 | rs2055232 | 8614880 | T | 0.3922 | 0.9984 | 0.9945 | none | 0.4953 | 1.51E-06 |
| 17 | . | 8615252 | T | 0.3921 | 0.9987 | 0.9957 |  | 0.4953 | 1.51E-06 |
| 17 | . | 8616815 | T | 0.3921 | 0.9974 | 0.9908 |  | 0.4953 | 1.51E-06 |
| 17 | . | 8616806 | R | 0.3921 | 0.9975 | 0.991 |  | 0.4953 | 1.51E-06 |
| 17 | rs4791742 | 8612403 | A | 0.4001 | 0.9785 | 0.9204 | none | 0.4972 | 1.76E-06 |
| 17 | . | 8612277 | R | 0.4002 | 0.9784 | 0.9196 |  | 0.4972 | 1.76E-06 |
| 8 | rs4739179 | 78785992 | G | 0.3495 | 0.991 | 0.9756 | none | 0.5026 | 3.04E-06 |
| 11 | rs1881509 | 1425605 | G | 0.4402 | 0.9572 | 0.8581 | *BRSK2* | 0.4934 | 3.48E-06 |
| 11 | rs10766750 | 1430916 | C | 0.438 | 0.9539 | 0.8483 | *BRSK2* | 0.4934 | 3.48E-06 |
| 17 | rs57025695 | 8619207 | C | 0.1626 | 0.9895 | 0.9397 | none | 0.4293 | 3.53E-06 |
| 17 | rs79170478 | 8611478 | C | 0.1695 | 0.9781 | 0.8665 | none | 0.4293 | 3.53E-06 |
| 17 | rs143866365 | 8612205 | A | 0.1696 | 0.979 | 0.8727 | none | 0.4293 | 3.53E-06 |
| 17 | rs148360444 | 8615101 | T | 0.1619 | 0.991 | 0.9475 | none | 0.4293 | 3.53E-06 |
| 8 | rs6983241 | 78783739 | G | 0.3495 | 0.9914 | 0.9768 | none | 0.5047 | 3.57E-06 |
| 8 | rs2631772 | 78767821 | T | 0.3579 | 0.9811 | 0.9347 | none | 0.5044 | 4.06E-06 |
| 8 | rs967210 | 78759124 | G | 0.3504 | 0.9963 | 0.9873 | none | 0.5085 | 4.12E-06 |
| 8 | rs1118738 | 78762134 | T | 0.3483 | 0.9938 | 0.9792 | none | 0.5085 | 4.12E-06 |
| 8 | rs1607540 | 78720149 | G | 0.3519 | 0.9993 | 0.9984 | none | 0.5085 | 4.12E-06 |
| 8 | rs1607541 | 78716497 | G | 0.3519 | 0.9993 | 0.9984 | none | 0.5085 | 4.12E-06 |
| 8 | rs1607542 | 78716459 | A | 0.3519 | 0.9992 | 0.9983 | none | 0.5085 | 4.12E-06 |
| 8 | rs1906169 | 78754292 | G | 0.3493 | 0.996 | 0.9863 | none | 0.5085 | 4.12E-06 |
| 8 | rs1979555 | 78755338 | A | 0.3507 | 0.9972 | 0.9906 | none | 0.5085 | 4.12E-06 |
| 8 | rs2054825 | 78756303 | T | 0.3492 | 0.9955 | 0.9846 | none | 0.5085 | 4.12E-06 |
| 8 | rs2339131 | 78762132 | T | 0.3484 | 0.9938 | 0.9792 | none | 0.5085 | 4.12E-06 |
| 8 | rs2631771 | 78755875 | A | 0.3508 | 0.997 | 0.9896 | none | 0.5085 | 4.12E-06 |
| 8 | rs2631773 | 78767792 | A | 0.3504 | 0.9943 | 0.981 | none | 0.5085 | 4.12E-06 |
| 8 | rs4546650 | 78717880 | C | 0.3519 | 0.9993 | 0.9984 | none | 0.5085 | 4.12E-06 |
| 8 | rs73235740 | 78733306 | T | 0.3517 | 0.999 | 0.997 | none | 0.5085 | 4.12E-06 |
| 8 | rs73693848 | 78741622 | C | 0.3537 | 0.9958 | 0.9861 | none | 0.5085 | 4.12E-06 |
| 8 | rs77518356 | 78730463 | A | 0.3501 | 0.9975 | 0.9908 | none | 0.5085 | 4.12E-06 |
| 8 | rs78631830 | 78723079 | G | 0.3519 | 0.9993 | 0.9981 | none | 0.5085 | 4.12E-06 |
| 8 | rs79306971 | 78723080 | T | 0.3502 | 0.9976 | 0.9912 | none | 0.5085 | 4.12E-06 |
| 8 | . | 78718310 | I | 0.3502 | 0.9976 | 0.9914 |  | 0.5085 | 4.12E-06 |
| 8 | . | 78755805 | I | 0.3512 | 0.9927 | 0.9719 |  | 0.5085 | 4.12E-06 |
| 11 | rs1574179 | 1421069 | G | 0.3986 | 0.9967 | 0.9872 | *BRSK2* | 0.5 | 4.14E-06 |
| 11 | rs4074036 | 1421703 | G | 0.4 | 0.996 | 0.9845 | *BRSK2* | 0.5 | 4.14E-06 |
| 11 | rs4963077 | 1423717 | C | 0.3994 | 0.9975 | 0.9919 | *BRSK2* | 0.5 | 4.14E-06 |
| 11 | rs7926201 | 1428780 | G | 0.3963 | 0.9973 | 0.9919 | *BRSK2* | 0.5 | 4.14E-06 |
| 11 | rs10766600 | 1419437 | G | 0.4013 | 0.9909 | 0.9641 | *BRSK2* | 0.5 | 4.14E-06 |
| 11 | rs11025492 | 1424342 | C | 0.3977 | 0.9987 | 0.9961 | *BRSK2* | 0.5 | 4.14E-06 |
| 11 | rs1881508 | 1420557 | C | 0.4425 | 0.9545 | 0.8484 | *BRSK2* | 0.4985 | 4.41E-06 |
| 11 | rs4312093 | 1421489 | G | 0.4415 | 0.9563 | 0.8548 | *BRSK2* | 0.4985 | 4.41E-06 |
| 11 | . | 1421138 | I | 0.4392 | 0.9544 | 0.847 |  | 0.4985 | 4.41E-06 |
| 17 | rs58064229 | 8620921 | T | 0.166 | 0.9875 | 0.9304 | none | 0.4337 | 4.50E-06 |
| 17 | rs60122827 | 8621386 | C | 0.1685 | 0.9845 | 0.9132 | none | 0.4337 | 4.50E-06 |
| 8 | rs10101178 | 78771909 | C | 0.3503 | 0.9933 | 0.9799 | none | 0.5107 | 4.81E-06 |
| 8 | rs1117935 | 78770113 | A | 0.345 | 0.9887 | 0.9619 | none | 0.509 | 4.89E-06 |
| 8 | rs1117937 | 78769805 | A | 0.345 | 0.9888 | 0.9621 | none | 0.509 | 4.89E-06 |
| 8 | rs1386337 | 78804682 | C | 0.348 | 0.9912 | 0.9721 | none | 0.5114 | 5.02E-06 |
| 8 | rs1486917 | 78808416 | T | 0.347 | 0.9918 | 0.9738 | none | 0.5114 | 5.02E-06 |
| 8 | rs2129462 | 78800995 | G | 0.3475 | 0.9915 | 0.9741 | none | 0.5114 | 5.02E-06 |
| 8 | rs2200557 | 78805782 | T | 0.3484 | 0.9922 | 0.9764 | none | 0.5114 | 5.02E-06 |
| 7 | rs78158938 | 36786796 | A | 0.039 | 0.9844 | 0.7069 | none | 0.2039 | 5.29E-06 |
| 8 | rs16939545 | 78635077 | A | 0.3443 | 0.964 | 0.8645 | none | 0.5112 | 5.71E-06 |
| 17 | rs57013214 | 8619060 | C | 0.1644 | 0.9901 | 0.9441 | none | 0.4381 | 5.72E-06 |
| 8 | rs1486924 | 78822850 | C | 0.3476 | 0.9938 | 0.9798 | none | 0.5136 | 5.87E-06 |
| 8 | rs1906171 | 78821736 | A | 0.3477 | 0.9937 | 0.9794 | none | 0.5136 | 5.87E-06 |
| 8 | rs2339196 | 78829443 | G | 0.3473 | 0.9946 | 0.9822 | none | 0.5136 | 5.87E-06 |
| 8 | rs4304321 | 78830146 | G | 0.3476 | 0.9944 | 0.9812 | none | 0.5136 | 5.87E-06 |
| 8 | rs4592020 | 78830133 | T | 0.3465 | 0.9943 | 0.9809 | none | 0.5136 | 5.87E-06 |
| 8 | rs4592021 | 78832656 | C | 0.3464 | 0.9946 | 0.9817 | none | 0.5136 | 5.87E-06 |
| 8 | rs7829327 | 78839452 | G | 0.3468 | 0.9948 | 0.9823 | none | 0.5136 | 5.87E-06 |
| 8 | . | 78825198 | T | 0.3476 | 0.994 | 0.9803 |  | 0.5136 | 5.87E-06 |
| 8 | rs10107592 | 78838775 | C | 0.347 | 0.995 | 0.9835 | none | 0.5136 | 5.87E-06 |
| 8 | rs10808822 | 78816889 | A | 0.3478 | 0.9934 | 0.9786 | none | 0.5136 | 5.87E-06 |
| 8 | . | 78816302 | R | 0.3479 | 0.9933 | 0.9784 |  | 0.5136 | 5.87E-06 |
| 11 | rs1554856 | 1433896 | T | 0.3943 | 0.9959 | 0.9893 | *BRSK2* | 0.5074 | 5.92E-06 |
| 8 | rs16939548 | 78641637 | T | 0.3478 | 0.9842 | 0.9401 | none | 0.5143 | 6.21E-06 |
| 8 | rs16939549 | 78642068 | A | 0.3507 | 0.9856 | 0.9451 | none | 0.5143 | 6.21E-06 |
| 8 | rs16939550 | 78642601 | T | 0.3505 | 0.9861 | 0.9471 | none | 0.5143 | 6.21E-06 |
| 8 | rs73232379 | 78636598 | A | 0.3483 | 0.9797 | 0.9216 | none | 0.5143 | 6.21E-06 |
| 8 | rs75363469 | 78637664 | T | 0.3486 | 0.9801 | 0.9235 | none | 0.5143 | 6.21E-06 |
| 8 | rs78721440 | 78639289 | C | 0.3486 | 0.9806 | 0.9252 | none | 0.5143 | 6.21E-06 |
| 8 | rs13248211 | 78792483 | G | 0.349 | 0.9914 | 0.976 | none | 0.515 | 6.47E-06 |
| 8 | rs1356678 | 78706571 | C | 0.3499 | 0.9974 | 0.9909 | none | 0.5174 | 6.75E-06 |
| 8 | rs1554347 | 78702588 | G | 0.3517 | 0.9991 | 0.9978 | none | 0.5174 | 6.75E-06 |
| 8 | rs2139328 | 78685275 | A | 0.3516 | 0.9997 | 0.9995 | none | 0.5174 | 6.75E-06 |
| 8 | rs7818316 | 78702131 | C | 0.3516 | 0.9992 | 0.9978 | none | 0.5174 | 6.75E-06 |
| 8 | rs7827238 | 78679395 | G | 0.3513 | 0.9994 | 0.9985 | none | 0.5174 | 6.75E-06 |
| 8 | rs9643727 | 78663612 | C | 0.3519 | 0.9995 | 0.999 | none | 0.5174 | 6.75E-06 |
| 8 | rs9643728 | 78664003 | G | 0.3515 | 0.9991 | 0.9974 | none | 0.5174 | 6.75E-06 |
| 8 | rs10504659 | 78686041 | A | 0.3513 | 0.9994 | 0.9985 | none | 0.5174 | 6.75E-06 |
| 8 | rs12676060 | 78655143 | T | 0.3512 | 0.9969 | 0.9885 | none | 0.5174 | 6.75E-06 |
| 8 | rs12677802 | 78699461 | G | 0.3516 | 0.9992 | 0.9978 | none | 0.5174 | 6.75E-06 |
| 8 | rs16939553 | 78649423 | A | 0.3508 | 0.9953 | 0.9816 | none | 0.5174 | 6.75E-06 |
| 8 | rs16939554 | 78651433 | C | 0.3512 | 0.9961 | 0.9851 | none | 0.5174 | 6.75E-06 |
| 8 | rs16939563 | 78679185 | G | 0.3495 | 0.9977 | 0.9914 | none | 0.5174 | 6.75E-06 |
| 8 | rs16939565 | 78682051 | G | 0.3516 | 0.9997 | 0.9996 | none | 0.5174 | 6.75E-06 |
| 8 | rs16939567 | 78696773 | C | 0.3505 | 0.9981 | 0.9933 | none | 0.5174 | 6.75E-06 |
| 8 | rs57436934 | 78691336 | G | 0.3517 | 0.9991 | 0.9973 | none | 0.5174 | 6.75E-06 |
| 8 | rs61405460 | 78658089 | G | 0.3515 | 0.9984 | 0.9946 | none | 0.5174 | 6.75E-06 |
| 8 | rs73232395 | 78656439 | A | 0.3514 | 0.9978 | 0.9919 | none | 0.5174 | 6.75E-06 |
| 8 | rs73233621 | 78666334 | C | 0.3515 | 0.9992 | 0.998 | none | 0.5174 | 6.75E-06 |
| 8 | rs73233625 | 78669481 | T | 0.3514 | 0.9991 | 0.9975 | none | 0.5174 | 6.75E-06 |
| 8 | rs73233630 | 78672871 | C | 0.3516 | 0.9992 | 0.9976 | none | 0.5174 | 6.75E-06 |
| 8 | rs73233633 | 78673355 | C | 0.3514 | 0.9993 | 0.9982 | none | 0.5174 | 6.75E-06 |
| 8 | rs73233634 | 78676882 | T | 0.3517 | 0.9996 | 0.9993 | none | 0.5174 | 6.75E-06 |
| 8 | rs73233640 | 78679881 | G | 0.3513 | 0.9994 | 0.9985 | none | 0.5174 | 6.75E-06 |
| 8 | rs111362187 | 78703651 | A | 0.3516 | 0.999 | 0.9973 | none | 0.5174 | 6.75E-06 |
| 8 | . | 78708042 | D | 0.3518 | 0.9991 | 0.9978 |  | 0.5174 | 6.75E-06 |
| 11 | rs1973846 | 1433696 | C | 0.3937 | 0.9925 | 0.9773 | *BRSK2* | 0.5094 | 6.86E-06 |
| 8 | rs2087135 | 78779389 | T | 0.3499 | 0.9924 | 0.9783 | none | 0.5165 | 7.25E-06 |
| 8 | rs10091510 | 78781745 | C | 0.3498 | 0.9922 | 0.9778 | none | 0.5165 | 7.25E-06 |
| 8 | rs12675830 | 78694682 | T | 0.3459 | 0.9949 | 0.9818 | none | 0.5189 | 7.44E-06 |
| 17 | rs59690594 | 8621415 | G | 0.1691 | 0.9832 | 0.9075 | none | 0.4438 | 8.06E-06 |
| 17 | rs61395178 | 8621452 | G | 0.1689 | 0.9833 | 0.9083 | none | 0.4438 | 8.06E-06 |
| 17 | rs111562727 | 8622745 | T | 0.1668 | 0.9847 | 0.9187 | none | 0.4438 | 8.06E-06 |
| 17 | rs139336244 | 8621669 | T | 0.1663 | 0.9857 | 0.9224 | none | 0.4438 | 8.06E-06 |
| 11 | rs7102279 | 1434680 | C | 0.3943 | 0.9958 | 0.9891 | *BRSK2* | 0.5148 | 8.40E-06 |
| 8 | rs9773896 | 78791108 | C | 0.348 | 0.9909 | 0.9736 | none | 0.5208 | 9.71E-06 |
| 11 | rs3934561 | 1424400 | C | 0.3797 | 0.9985 | 0.9949 | *BRSK2* | 0.5152 | 9.82E-06 |
| 11 | rs4074035 | 1421716 | C | 0.3792 | 0.9963 | 0.9857 | *BRSK2* | 0.5152 | 9.82E-06 |
| 11 | rs4963041 | 1423770 | G | 0.3789 | 0.9979 | 0.9924 | *BRSK2* | 0.5152 | 9.82E-06 |
| 11 | rs4963076 | 1423176 | T | 0.3805 | 0.9996 | 0.9994 | *BRSK2* | 0.5152 | 9.82E-06 |
| 11 | rs7103987 | 1422794 | C | 0.3795 | 0.9979 | 0.992 | *BRSK2* | 0.5152 | 9.82E-06 |
| 11 | rs10833257 | 1422726 | T | 0.3795 | 0.9978 | 0.9917 | *BRSK2* | 0.5152 | 9.82E-06 |
| 1 | rs1417150 | 203196757 | T | 0.0946 | 0.9636 | 0.661 | *CHIT1* | 0.3519 | 1.08E-05 |
| 17 | rs1004445 | 8645367 | A | 0.499 | 0.9968 | 0.9894 | *CCDC42* | 0.5293 | 1.15E-05 |
| 17 | rs9894347 | 8646158 | C | 0.4984 | 0.9993 | 0.9983 | *CCDC42* | 0.5293 | 1.15E-05 |
| 4 | rs2902922 | 9609380 | G | 0.0183 | 0.989 | 0.5499 | none | 0.1327 | 1.21E-05 |
| 4 | rs9917891 | 9614633 | C | 0.017 | 0.9859 | 0.4351 | none | 0.1327 | 1.21E-05 |
| 4 | rs9917943 | 9612413 | C | 0.0183 | 0.9876 | 0.5016 | none | 0.1327 | 1.21E-05 |
| 4 | rs9917966 | 9614537 | G | 0.017 | 0.986 | 0.436 | none | 0.1327 | 1.21E-05 |
| 4 | rs58673888 | 9601558 | T | 0.0179 | 0.9916 | 0.651 | none | 0.1327 | 1.21E-05 |
| 4 | rs58820969 | 9607071 | A | 0.0168 | 0.9913 | 0.6354 | none | 0.1327 | 1.21E-05 |
| 4 | rs112013141 | 9611339 | G | 0.0186 | 0.9883 | 0.5274 | none | 0.1327 | 1.21E-05 |
| 4 | rs116345678 | 9601756 | G | 0.0178 | 0.9916 | 0.6511 | none | 0.1327 | 1.21E-05 |
| 4 | rs116386123 | 9593389 | A | 0.0164 | 0.9873 | 0.4813 | none | 0.1327 | 1.21E-05 |
| 4 | rs137952419 | 9613766 | G | 0.0164 | 0.9857 | 0.4211 | none | 0.1327 | 1.21E-05 |
| 4 | rs144989810 | 9612047 | T | 0.0159 | 0.9889 | 0.5362 | none | 0.1327 | 1.21E-05 |
| 20 | rs6022774 | 52431105 | A | 0.486 | 0.9652 | 0.8903 | none | 0.5164 | 1.30E-05 |
| 17 | rs60521331 | 8639905 | A | 0.4764 | 0.9777 | 0.9313 | *CCDC42* | 0.5383 | 1.74E-05 |
| 8 | rs2339132 | 78770532 | T | 0.4605 | 0.9921 | 0.976 | none | 0.5441 | 1.75E-05 |
| 3 | rs1532428 | 82969840 | G | 0.3139 | 0.9994 | 0.9995 | none | 0.5295 | 1.99E-05 |
| 3 | rs17422129 | 82969622 | C | 0.3136 | 0.999 | 0.9976 | none | 0.5295 | 1.99E-05 |
| 11 | rs1881510 | 1425910 | T | 0.3426 | 0.9549 | 0.8491 | *BRSK2* | 0.5235 | 2.04E-05 |
| 20 | rs6095947 | 49061430 | C | 0.4909 | 0.9621 | 0.8843 | none | 1.888 | 2.06E-05 |
| 20 | rs6095948 | 49061666 | T | 0.4909 | 0.9617 | 0.8828 | none | 1.888 | 2.06E-05 |
| 20 | rs6095949 | 49061728 | G | 0.491 | 0.9614 | 0.8816 | none | 1.888 | 2.06E-05 |
| 1 | rs1002485 | 203189488 | T | 0.0874 | 0.9768 | 0.786 | *CHIT1* | 0.3679 | 2.14E-05 |
| 1 | rs1556854 | 203189552 | T | 0.0874 | 0.9769 | 0.787 | *CHIT1* | 0.3679 | 2.14E-05 |
| 1 | rs2486962 | 203191920 | G | 0.0889 | 0.9781 | 0.8025 | *CHIT1* | 0.3679 | 2.14E-05 |
| 1 | rs2486963 | 203194008 | A | 0.0862 | 0.9801 | 0.8226 | *CHIT1* | 0.3679 | 2.14E-05 |
| 1 | rs2486069 | 203193736 | C | 0.1124 | 0.9995 | 0.9957 | *CHIT1* | 0.4081 | 2.17E-05 |
| 8 | rs936890 | 78864140 | A | 0.337 | 0.9924 | 0.9747 | none | 0.535 | 2.20E-05 |
| 17 | rs9906231 | 8636265 | G | 0.4869 | 0.9783 | 0.9335 | *CCDC42* | 0.5458 | 2.37E-05 |
| 17 | rs35490467 | 8637221 | C | 0.487 | 0.979 | 0.9352 | *CCDC42* | 0.5458 | 2.37E-05 |
| 17 | rs35742952 | 8636832 | G | 0.4869 | 0.9785 | 0.9341 | *CCDC42* | 0.5458 | 2.37E-05 |
| 2 | rs13426854 | 240845694 | T | 0.0362 | 0.9774 | 0.5243 | none | 0.2076 | 2.39E-05 |
| 20 | rs4809937 | 52426802 | C | 0.4869 | 0.9722 | 0.9167 | none | 1.891 | 2.50E-05 |
| 8 | rs2339134 | 78860395 | T | 0.3381 | 0.9925 | 0.9742 | none | 0.5374 | 2.56E-05 |
| 8 | rs6982507 | 78849410 | G | 0.34 | 0.9912 | 0.969 | none | 0.5374 | 2.56E-05 |
| 8 | . | 78851274 | R | 0.3383 | 0.9928 | 0.9747 |  | 0.5374 | 2.56E-05 |
| 8 | . | 78864811 | R | 0.3382 | 0.9923 | 0.9739 |  | 0.5374 | 2.56E-05 |
| 8 | . | 78817310 | R | 0.3642 | 0.9897 | 0.9646 |  | 0.5419 | 2.73E-05 |
| 18 | rs8085967 | 52654114 | A | 0.0866 | 0.9676 | 0.71 | none | 0.3809 | 2.74E-05 |
| 20 | rs6095950 | 49062165 | T | 0.4894 | 0.9604 | 0.8782 | none | 1.867 | 2.74E-05 |
| 20 | rs6095951 | 49062284 | G | 0.4892 | 0.9603 | 0.878 | none | 1.867 | 2.74E-05 |
| 20 | rs6063515 | 49056905 | G | 0.4916 | 0.9597 | 0.876 | none | 1.866 | 2.84E-05 |
| 20 | rs6067449 | 49056840 | T | 0.492 | 0.9591 | 0.874 | none | 1.866 | 2.84E-05 |
| 11 | rs10833729 | 1443748 | G | 0.3856 | 0.9875 | 0.9501 | *BRSK2* | 0.5374 | 2.87E-05 |
| 10 | rs7916242 | 54048234 | G | 0.38 | 0.9985 | 0.996 | *PRKG1* | 0.5439 | 2.98E-05 |
| 20 | rs6063517 | 49059241 | A | 0.4916 | 0.9622 | 0.8847 | none | 1.857 | 3.04E-05 |
| 20 | rs6095946 | 49060191 | C | 0.4916 | 0.9633 | 0.8884 | none | 1.857 | 3.04E-05 |
| 20 | rs7273775 | 49061320 | C | 0.4916 | 0.9632 | 0.8883 | none | 1.857 | 3.04E-05 |
| 20 | rs8117653 | 49060648 | T | 0.4912 | 0.9634 | 0.8889 | none | 1.857 | 3.04E-05 |
| 11 | rs4881746 | 1440375 | C | 0.3825 | 0.9878 | 0.9513 | *BRSK2* | 0.5426 | 3.55E-05 |
| 11 | rs4963078 | 1435329 | A | 0.385 | 0.9997 | 0.9998 | *BRSK2* | 0.5426 | 3.55E-05 |
| 11 | rs4963079 | 1435379 | T | 0.3849 | 0.9998 | 1 | *BRSK2* | 0.5426 | 3.55E-05 |
| 11 | rs4963080 | 1435853 | G | 0.3861 | 0.9965 | 0.9863 | *BRSK2* | 0.5426 | 3.55E-05 |

**S2 Tables B (i)**: Results of *in silico* replication of results for markers in *BRSK2* using summary data from a GWAS in opioid dependence in two different populations, African American and European American.

|  |  |  |  | **HAN CHINESE** | | | | **AFRICAN AMERICAN** | | | | **EUROPEAN AMERICAN** | | | |
| --- | --- | --- | --- | --- | --- | --- | --- | --- | --- | --- | --- | --- | --- | --- | --- |
| **CHR** | **Gene** | **SNP** | **BP** | **A1** | **MAF** | **OR** | **P** | **A1** | **MAF** | **OR** | **P** | **A1** | **MAF** | **OR** | **P** |
| 11 | ***BRSK2*** | rs10766600 | 1419437 | G | 0.40 | 0.50 | 4.14E-06 | A | 0.32 | 1.03 | 0.7602 | A | 0.24 | 0.97 | 0.7352 |
| 11 | ***BRSK2*** | rs1881508 | 1420557 | C | 0.44 | 0.50 | 4.41E-06 | T | 0.08 | 0.91 | 0.5311 | T | 0.19 | 0.91 | 0.4194 |
| 11 | ***BRSK2*** | rs1574179 | 1421069 | G | 0.40 | 0.50 | 4.14E-06 | A | 0.34 | 1.02 | 0.8199 | A | 0.24 | 0.96 | 0.6952 |
| 11 | ***BRSK2*** | rs4312093 | 1421489 | G | 0.44 | 0.50 | 4.41E-06 | A | 0.08 | 0.89 | 0.4801 | A | 0.19 | 0.90 | 0.3847 |
| 11 | ***BRSK2*** | rs4074036 | 1421703 | G | 0.40 | 0.50 | 4.14E-06 | A | 0.34 | 1.02 | 0.8168 | A | 0.24 | 0.96 | 0.6955 |
| 11 | ***BRSK2*** | rs4074035 | 1421716 | C | 0.38 | 0.52 | 9.82E-06 | C | 0.32 | 1.14 | 0.1138 | C | 0.47 | 0.87 | 0.1280 |
| 11 | ***BRSK2*** | rs10833257 | 1422726 | T | 0.38 | 0.52 | 9.82E-06 | T | 0.32 | 1.14 | 0.1157 | T | 0.47 | 0.88 | 0.1628 |
| 11 | ***BRSK2*** | rs7103987 | 1422794 | C | 0.38 | 0.52 | 9.82E-06 | C | 0.32 | 1.14 | 0.1159 | C | 0.47 | 0.88 | 0.1625 |
| 11 | ***BRSK2*** | rs4963076 | 1423176 | T | 0.38 | 0.52 | 9.82E-06 | T | 0.32 | 1.13 | 0.1330 | C | 0.50 | 0.87 | 0.1363 |
| 11 | ***BRSK2*** | rs4963077 | 1423717 | C | 0.40 | 0.50 | 4.14E-06 | T | 0.34 | 1.02 | 0.8129 | T | 0.24 | 0.96 | 0.6966 |
| 11 | ***BRSK2*** | rs4963041 | 1423770 | G | 0.38 | 0.52 | 9.82E-06 | G | 0.32 | 1.13 | 0.1267 | G | 0.47 | 0.88 | 0.1607 |
| 11 | ***BRSK2*** | rs11025492 | 1424342 | C | 0.40 | 0.50 | 4.14E-06 | T | 0.33 | 1.02 | 0.8329 | T | 0.24 | 0.96 | 0.6964 |
| 11 | ***BRSK2*** | rs3934561 | 1424400 | C | 0.38 | 0.52 | 9.82E-06 | C | 0.36 | 1.14 | 0.1133 | T | 0.50 | 0.87 | 0.1328 |
| 11 | ***BRSK2*** | rs1881509 | 1425605 | G | 0.44 | 0.49 | 3.48E-06 | A | 0.05 | 0.85 | 0.4299 | A | 0.19 | 0.91 | 0.3858 |
| 11 | ***BRSK2*** | rs1881510 | 1425910 | T | 0.34 | 0.52 | 2.04E-05 | C | 0.46 | 0.94 | 0.4007 | C | 0.34 | 1.03 | 0.7829 |
| 11 | ***BRSK2*** | rs7926201 | 1428780 | G | 0.40 | 0.50 | 4.14E-06 | A | 0.34 | 1.02 | 0.8359 | A | 0.24 | 0.96 | 0.6882 |
| 11 | ***BRSK2*** | rs10766750 | 1430916 | C | 0.44 | 0.49 | 3.48E-06 | T | 0.05 | 0.84 | 0.4035 | T | 0.19 | 0.90 | 0.3796 |
| 11 | ***BRSK2*** | rs1973846 | 1433696 | C | 0.39 | 0.51 | 6.86E-06 | T | 0.35 | 1.07 | 0.3788 | T | 0.26 | 0.96 | 0.6607 |
| 11 | ***BRSK2*** | rs1554856 | 1433896 | T | 0.39 | 0.51 | 5.92E-06 | G | 0.34 | 1.01 | 0.8930 | G | 0.24 | 0.96 | 0.7038 |
| 11 | ***BRSK2*** | rs7102279 | 1434680 | C | 0.39 | 0.51 | 8.40E-06 | T | 0.34 | 1.02 | 0.8541 | T | 0.24 | 0.96 | 0.6840 |
| 11 | ***BRSK2*** | rs4963078 | 1435329 | A | 0.39 | 0.54 | 3.55E-05 | G | 0.34 | 1.00 | 0.9634 | G | 0.24 | 0.97 | 0.7319 |
| 11 | ***BRSK2*** | rs4963079 | 1435379 | T | 0.38 | 0.54 | 3.55E-05 | C | 0.34 | 1.00 | 0.9597 | C | 0.24 | 0.95 | 0.6509 |
| 11 | ***BRSK2*** | rs4963080 | 1435853 | G | 0.39 | 0.54 | 3.55E-05 | A | 0.34 | 1.00 | 0.9646 | A | 0.24 | 0.97 | 0.7315 |
| 11 | ***BRSK2*** | rs4881746 | 1440375 | C | 0.38 | 0.54 | 3.55E-05 | T | 0.35 | 0.99 | 0.9171 | T | 0.26 | 0.92 | 0.4108 |
| 11 | ***BRSK2*** | rs10833729 | 1443748 | G | 0.39 | 0.54 | 2.87E-05 | A | 0.34 | 1.00 | 0.9914 | A | 0.24 | 0.95 | 0.5943 |

**S2 Table B (ii):** *In silico* replication of results for markers in and around the top gene, *CCDC42*, using summary data from a GWAS in opioid dependence in African American and European American populations

|  |  |  |  | **HAN CHINESE** | | | | **AFRICAN AMERICAN** | | | | **EUROPEAN AMERICAN** | | | |
| --- | --- | --- | --- | --- | --- | --- | --- | --- | --- | --- | --- | --- | --- | --- | --- |
| **CHR** | **Gene** | **SNP** | **BP** | **A1** | **MAF** | **OR** | **P** | A1 | **MAF** | **OR** | **P** | **A1** | **MAF** | **OR** | **P** |
| 17 | none | rs7219393 | 8617731 | A | 0.38 | 0.49 | 8.98E-07 | A | 0.24 | 0.98 | 0.8583 | A | 0.22 | 1.06 | 0.5854 |
| 17 | none | rs4275897 | 8620609 | A | 0.39 | 0.49 | 1.19E-06 | A | 0.24 | 0.99 | 0.9148 | A | 0.22 | 1.06 | 0.5881 |
| 17 | none | rs7219141 | 8626010 | A | 0.39 | 0.47 | 3.36E-07 | A | 0.41 | 0.95 | 0.4935 | A | 0.22 | 1.06 | 0.5641 |
| 17 | none | rs4791746 | 8626357 | T | 0.39 | 0.46 | 2.20E-07 | T | 0.26 | 1.00 | 0.9643 | T | 0.22 | 1.07 | 0.5386 |
| 17 | none | rs4791747 | 8626695 | A | 0.39 | 0.46 | 2.20E-07 | A | 0.25 | 1.02 | 0.8226 | A | 0.22 | 1.07 | 0.5342 |
| 17 | none | rs5019589 | 8626803 | A | 0.16 | 0.40 | 8.47E-07 | A | 0.05 | 0.98 | 0.9171 | A | 0.03 | . | . |
| 17 | none | rs5019588 | 8626857 | T | 0.39 | 0.46 | 2.20E-07 | T | 0.34 | 0.96 | 0.6237 | T | 0.43 | 1.13 | 0.2299 |
| 17 | none | rs7215874 | 8628632 | T | 0.39 | 0.48 | 6.99E-07 | T | 0.41 | 0.96 | 0.6267 | T | 0.22 | 1.06 | 0.5553 |
| 17 | none | rs9303230 | 8630396 | G | 0.39 | 0.47 | 3.71E-07 | G | 0.40 | 0.96 | 0.6401 | G | 0.22 | 1.06 | 0.5610 |
| 17 | none | rs9915929 | 8630420 | A | 0.39 | 0.47 | 3.71E-07 | A | 0.40 | 0.96 | 0.6404 | A | 0.22 | 1.06 | 0.5611 |
| 17 | none | rs4791326 | 8631788 | G | 0.39 | 0.47 | 3.71E-07 | G | 0.25 | 1.03 | 0.7270 | G | 0.22 | 1.07 | 0.5240 |
| 17 | ***CCDC42*** | rs112414721 | 8633714 | A | 0.17 | 0.41 | 1.02E-06 | A | 0.14 | 0.97 | 0.7644 | A | 0.03 | . | . |
| 17 | ***CCDC42*** | rs9906231 | 8636265 | G | 0.49 | 0.55 | 2.37E-05 | C | 0.50 | 0.87 | 0.0936 | G | 0.46 | 0.96 | 0.6733 |
| 17 | ***CCDC42*** | rs35742952 | 8636832 | G | 0.49 | 0.55 | 2.37E-05 | G | 0.50 | 0.87 | 0.0944 | G | 0.46 | 0.96 | 0.6758 |
| 17 | ***CCDC42*** | rs35490467 | 8637221 | C | 0.49 | 0.55 | 2.37E-05 | T | 0.50 | 0.87 | 0.0948 | C | 0.46 | 0.96 | 0.6780 |
| 17 | ***CCDC42*** | rs58558356 | 8639656 | A | 0.17 | 0.40 | 6.53E-07 | A | 0.15 | 0.95 | 0.6442 | A | 0.03 | 1.14 | 0.6178 |
| 17 | ***CCDC42*** | rs60521331 | 8639905 | A | 0.48 | 0.54 | 1.74E-05 | A | 0.36 | 0.96 | 0.6002 | A | 0.44 | 0.92 | 0.3215 |
| 17 | ***CCDC42*** | rs73977403 | 8640012 | A | 0.17 | 0.40 | 6.53E-07 | A | 0.15 | 0.95 | 0.6798 | A | 0.03 | . | . |
| 17 | ***CCDC42*** | rs80051038 | 8641573 | T | 0.17 | 0.40 | 5.05E-07 | C | 0.04 | 0.98 | 0.9306 | T | 0.03 | . | . |
| 17 | ***CCDC42*** | rs60363471 | 8642441 | C | 0.17 | 0.40 | 5.05E-07 | G | 0.04 | 0.98 | 0.9229 | C | 0.03 | . | . |
| 17 | ***CCDC42*** | rs116694173 | 8642642 | T | 0.17 | 0.40 | 5.05E-07 | C | 0.04 | 0.98 | 0.9211 | T | 0.03 | . | . |
| 17 | ***CCDC42*** | rs2288156 | 8644854 | T | 0.17 | 0.39 | 3.48E-07 | C | 0.04 | 0.97 | 0.8743 | T | 0.03 | . | . |
| 17 | ***CCDC42*** | rs1004445 | 8645367 | A | 0.50 | 0.53 | 1.15E-05 | G | 0.43 | 0.96 | 0.5870 | A | 0.46 | 0.95 | 0.5889 |
| 17 | ***CCDC42*** | rs9894347 | 8646158 | C | 0.50 | 0.53 | 1.15E-05 | T | 0.33 | 0.95 | 0.5315 | C | 0.46 | 0.96 | 0.6791 |

**S2 Table C (i)**: Table showing common candidate genes previously tested in Chinese samples. The table shows the top result in these published studies and the type of polymorphism yielding the result. This list was used to assess gene-level replication in our data.

| **GENE** | **STUDY** | **POLYMORPHISM** | **RESULTS of association analysis** |
| --- | --- | --- | --- |
| ***OPRM1*** | Szeto et al, 2001 | A118G | G allele associated with heroin use; p=0.016 |
|  |  | C1031G | G allele associated with heroin use; p=0.014 |
|  |  |  |  |
|  | Shi et al, 2002 | A118G | No association |
|  |  | G31A | G31A associated with higher intake of heroin |
|  |  | G877A | No association |
|  |  | G77A | No association |
|  |  | C9T-3' UTR | No association |
|  |  |  |  |
|  | Li et al, 2000 | A118G | No association |
|  |  | G691C | No association |
|  |  |  |  |
| ***OPRD1*** | Xu et al, 2002 | T921C | No association |
|  |  |  |  |
| ***DRD2*** | Li et al, 2002 | Taq1A1 | No association |
|  |  | Ser311Cys | No association |
|  |  | promoter -141ΔC | Association observed with subset of subjects, who inhaled heroin as opposed to injecting; p=0.006 |
|  |  |  |  |
|  | Xu et al, 2004 | 10 SNPs | No association with single SNPs; association observed with haplotype comprised of 8 SNPs; p=1.425x10^-22^ |
|  |  |  |  |
|  | Hou and Li, 2009 | Taq1A1 (rs1800497) | heroin dependence: p=0.014 |
|  |  |  |  |
| ***DRD3*** | Li et al, 2002 | Ser9Gly | No association |
|  |  |  |  |
| ***DRD4*** | Shao et al, 2006 | VNTR, exon 3 | Association between long repeats and craving: p=0.012 |
|  |  |  |  |
|  | Li et al, 1997 | VNTR, exon 3 | Long repeats and abuse: p=0.023 |
|  | Li et al, 2000 | VNTR, exon 3; 521C/T | VNTR associated with inhalers: p=0.002 |
|  |  |  |  |
| ***DAT (SLC6A3)*** | Hou and Li, 2009 | VNTR | No association |
|  |  |  |  |
| ***GABRB2*** | Loh et al, 2007 | rs2229944 | No association |
|  |  |  |  |
| ***GABRA6*** | Loh et al, 2207 | rs3219151 | No association |
|  |  |  |  |
| ***GABRA1*** | Loh et al, 2007 | rs2279020 | No association |
|  |  |  |  |
| ***GABRG2*** | Loh et al, 2007 | rs4480617 | No association |
|  |  | rs211013 | No association |
|  |  | rs211014 | p=0.015 |
|  |  |  |  |
| ***GABRG2*** | Li et al, 2002 | 3145G/A | No association |
|  |  |  |  |
| ***CNR1*** | Li et al, 2000 | Triplet repeat (AAT) | No association |
|  |  |  |  |
| ***HTR1B*** | Gao et al, 2011 | G861C | G allele associated with dependence; p=001 |
|  |  | A1180G | No association |
|  |  |  |  |
| ***HTR2A*** | Li et al, 2002 | Promoter 1438G/A | No association |
|  |  | T102C | No association |
|  | Gao et al, 2011 | A-1438G | No association |
|  |  | T102C | No association |
|  |  |  |  |
| ***5-HTT (SLC6A4)*** | Li et al, 2002 | VNTR, intron 2 | No association |
|  |  | Insertion/Del in promoter | No association |
|  |  |  |  |

**S2 Table C (ii)**: Gene-based replication using the list of genes compiled from previous published reports, as shown in Table S3a (above) and assessing their association status in the results from the VEGAS2 analysis performed in the current study.

| **Chr** | **Gene** | **nSNPs** | **Pvalue** | **TopSNP** | **TopSNP-pvalue** |
| --- | --- | --- | --- | --- | --- |
|  |  |  |  |  |  |
| 6 | OPRM1 | 495 | 0.9590 | rs6930716 | 0.0711 |
| 1 | OPRD1 | 179 | 0.1229 | rs525212 | 0.0306 |
| 11 | DRD2 | 147 | 0.3497 | rs60023970 | 0.0125 |
| 3 | DRD3 | 249 | 0.4965 | rs12487548 | 0.0101 |
| 11 | DRD4 | 128 | 0.7253 | rs79183202 | 0.0215 |
| 5 | SLC6A3 | 159 | 0.4895 | rs62331136 | 0.0680 |
| 5 | GABRB2 | 561 | 0.8551 | rs75820188 | 0.0162 |
| 5 | GABRA6 | 232 | 0.1588 | rs12186598 | 0.0119 |
| 5 | GABRA1 | 234 | 0.2398 | rs10057566 | 0.0353 |
| 5 | GABRG2 | 148 | 0.8661 | rs10462930 | 0.0283 |
| 6 | HTR1B | 178 | 0.8981 | rs1778255 | 0.1081 |
| 13 | HTR2A | 222 | 0.9101 | rs112452802 | 0.2083 |
| 17 | SLC6A4 | 124 | 0.0413 | rs188181203 | 0.0079 |
|  |  |  |  |  |  |

**S2 Table D**: Top ten results of the pathway analysis using the MSigDB database. The p-value calculated for each pathway was adjusted using the Benjamini and Hochberg method to yield an empirical value. The number of genes in each pathway are also noted.

| **SELF_P** | **FDR-adjusted p-value (q-value)** | **NAME OF PATHWAY** | **NGENES** |
| --- | --- | --- | --- |
|  |  |  |  |
| 0.30644 | 0.576 | GO: REGULATION OF VACUOLAR TRANSPORT | 25 |
| 0.03048 | 0.576 | GO: REGULATION OF SKELETAL MUSCLE CONTRACTION | 10 |
| 0.02398 | 0.576 | REACTOME: FATTY ACYL COA BIOSYNTHESIS | 17 |
| 0.04385 | 0.576 | GO: POSITIVE REGULATION OF NUCLEAR DIVISION | 60 |
| 0.00347 | 0.576 | KEGG: DORSO VENTRAL AXIS FORMATION | 24 |
| 0.02598 | 0.576 | GO: NEGATIVE REGULATION OF SKELETAL MUSCLE TISSUE DEVELOPMENT | 11 |
| 0.22963 | 0.576 | GO: POSITIVE REGULATION OF VACUOLAR TRANSPORT | 13 |
| 0.04810 | 0.618 | GO: POSITIVE REGULATION OF METAPHASE ANAPHASE TRANSITION OF CELL CYCLE | 14 |
| 0.54340 | 0.618 | GO: POSITIVE REGULATION OF SODIUM ION TRANSMEMBRANE TRANSPORT | 14 |
| 0.03532 | 0.618 | GO: GLUCURONOSYLTRANSFERASE ACTIVITY | 35 |
|  |  |  |  |
